# The causal effect of physical activity intensity on COVID-19 susceptibility, hospitalization, and severity: Evidence from a mendelian randomization study

**DOI:** 10.3389/fphys.2023.1089637

**Published:** 2023-03-08

**Authors:** Xing Zhang, Xinyue Zhang, Siyuan Feng, Hansen Li

**Affiliations:** ^1^ Institute of Sports Science, College of Physical Education, Southwest University, Chongqing, China; ^2^ Graduate School, University of Wisconsin-Madison, Madison, WI, United States; ^3^ Laboratory of Genetics, University of Wisconsin-Madison, Madison, WI, United States

**Keywords:** COVID-19, public health, sports science, exercise, individual treatment

## Abstract

The protection of physical activity (PA) against COVID-19 is a rising research interest. However, the role of physical activity intensity on this topic is yet unclear. To bridge the gap, we performed a Mendelian randomization (MR) study to verify the causal influence of light and moderate-to-vigorous PA on COVID-19 susceptibility, hospitalization, and severity. The Genome-Wide Association Study (GWAS) dataset of PA (*n* = 88,411) was obtained from the UK biobank and the datasets of COVID-19 susceptibility (*n* = 1,683,768), hospitalization (*n* = 1,887,658), and severity (*n* = 1,161,073) were extracted from the COVID-19 Host Genetics Initiative. A random-effect inverse variance weighted (IVW) model was carried out to estimate the potential causal effects. A Bonferroni correction was used for counteracting. The problem of multiple comparisons. MR-Egger test, MR-PRESSO test, Cochran’s Q statistic, and Leave-One-Out (LOO) were used as sensitive analysis tools. Eventually, we found that light PA significantly reduced the risk of COVID-19 infection (OR = 0.644, 95% CI: 0.480–0.864, *p* = 0.003). Suggestive evidence indicated that light PA reduced the risks of COVID-19 hospitalization (OR = 0.446, 95% CI: 0.227 to 0.879, *p* = 0.020) and severe complications (OR = 0.406, 95% CI: 0.167–0.446, *p* = 0.046). By comparison, the effects of moderate-to-vigorous PA on the three COVID-19 outcomes were all non-significant. Generally, our findings may offer evidence for prescribing personalized prevention and treatment programs. Limited by the available datasets and the quality of evidence, further research is warranted to re-examine the effects of light PA on COVID-19 when new GWAS datasets emerge.

## 1 Introduction

Coronavirus disease 19 (COVID-19) is an infectious disease first detected in December 2019, which is caused by the severe acute respiratory syndrome coronavirus-2 (SARS-CoV-2) virus (Yuki et al., 2020; Chen Y. et al., 2022). This virus is primarily transmitted *via* airborne routes, making it highly infectious and thereby a global pandemic (Greenhalgh et al., 2021). According to the Center for Systems Science and Engineering at Johns Hopkins University, the pandemic has caused over 623.9 million confirmed cases and over 6.5 million confirmed deaths by 14 October 2022 (Weber et al., 2016). Vaccine injection is currently the most effective strategy against the pandemic (Chen Y. et al., 2022). However, the vaccine’s effectiveness appears to be unsatisfactory owing to the SARS-CoV-2 mutation, such as the Omicron variant (Abdool Karim and de Oliveira, 2021; Zhang M. et al., 2022; Singanayagam et al., 2022). Given that, identifying behaviorally protective factors in mitigating COVID-19 is necessary for prescribing personalized treatment programs and reasonably allocating public health resources (Chen X. et al., 2022).

Physical activity (PA) is one of the most popular strategies for public health promotion (Zhang et al., 2022a; Zhang et al., 2022b). In the past decades, PA has been widely implemented to cope with various health problems, such as cardiovascular disease (Wannamethee and Shaper, 2001), Alzheimer’s disease (Scarmeas et al., 2009), and most importantly, upper-respiratory tract disease (Matthews et al., 2002). These underscore the potential of PA in the prevention and treatment of COVID-19. For instance, observational studies have implied that PA is associated with a lowered risk of COVID-19 hospitalization, severity, and mortality (Hamer et al., 2020; Steenkamp et al., 2022). However, due to the inherent defects of traditional observational studies that the possibility of reverse causality and confounding factors cannot be entirely excluded (Sekula et al., 2016), the efficacy of PA in preventing COVID-19 remains unclear.

Mendelian randomization (MR), by contrast, is an ideal approach to cope with the defects described above. MR can effectively preclude confounding factors and uncover causal relationships by using genetic variants randomly allocated conception to proxy exposure (Davey Smith and Hemani, 2014; Davies et al., 2018). So far, several MR studies have been performed to check the effect of PA on COVID-19 outcomes. Zhang et al. (2020) found that some PA variables (except for moderate-to-vigorous PA) lowered the COVID-19 infection and outpatient, and similar findings were reported elsewhere (Chen X. et al., 2022). These studies collectively indicate that the effect of PA on COVID-19 outcomes may vary with intensity. Nevertheless, the other ranges of PA intensity, such as light PA, are understudied. Accordingly, the current MR study aimed to examine the causal effects of light and moderate-to-vigorous PA on COVID-19 susceptibility, hospitalization, and severity.

## 2 Materials and methods

### 2.1 Study design

In the current study, a two-sample Mendelian randomization design was selected to estimate the potential causal influence of light and moderate-to-vigorous PA on COVID-19 susceptibility, hospitalization, and severity. Single nucleotide polymorphisms (SNPs) were used as instrumental variables to exclude confounding factors and infer causality. To control population stratification bias, we kept mainly individuals of European ancestry for the current analyses (Lin et al., 2022). Our study design is as follows:

(1) SNPs associated with exposure (light and moderate-to-vigorous PA) were identified at the genome-wide significance threshold;
(2) SNPs are independent of potential confounders;
(3) SNPs affect COVID-19 only *via* exposure.

### 2.2 Data sources and single-nucleotide polymorphisms selection

#### 2.2.1 Physical activity

Genome-wide association study (GWAS) data for accelerometer-measured PA was obtained from the UK Biobank (http://www.nealelab.is/uk-biobank) (Qi et al., 2022). The GWAS data involve 88,411 European ancestry participants (Qi et al., 2022). The GWAS data were adjusted for age, sex, and the first 20 genetic principal components (Qi et al., 2022). Two PA phenotypes were included in the current MR analysis as exposures, including light and moderate-to-vigorous intensity. Light PA was defined as the duration during which the acceleration was at least 30 mg (Milli-gravity) but lower than 100 mg. On the other hand, moderate-to-vigorous PA was defined as the duration during which the acceleration was at least 100 mg. The SNPs were selected by the following criteria: 1) SNPs associated with accelerometer-measured PA were identified at the genome-wide significance threshold (*p* < 5 × 10^−7^) (Kanai et al., 2016); 2) SNPs without linkage disequilibrium (*r* ^2^ < 0.01 and clump window <10 MB) (Yuan et al., 2022); 3) SNPs without potential pleiotropic effects (Bahls et al., 2021); and 4) SNPs having F-statistic >10 was considered evidence of valid instrumental variables and were excluded from MR analysis (Papadimitriou et al., 2021; Ren et al., 2022).

Finally, seven SNPs were used as instrumental variables (IVs) for light PA (Supplementary Table S1), and five SNPs were used for moderate-to-vigorous PA (Supplementary Table S2).

#### 2.2.2 COVID-19 outcomes

The GWAS datasets for COVID-19 outcomes were obtained from the COVID-19 Host Genetics Initiative, which is an international genetics collaboration aiming to discover the genetic determinants of COVID-19 and its consequences (COVID19-HGI, 2021; Leong et al., 2021). The GWAS datasets were adjusted for age, age^2^, sex, age × sex, principal components, and study-specific covariates by the original GWAS investigators (Yeung et al., 2022). Three COVID-19 phenotypes were included in the current study as outcomes, including susceptibility, hospitalization, and severity. The GWAS dataset of COVID-19 susceptibility compares COVID-19 cases (*n* = 38,984) with population controls (*n* = 1,644,784). In the dataset, COVID-19 cases are defined as laboratory-confirmed SARS-CoV-2 positive from electronic health records or doctor diagnoses or self-reported (Chen X. et al., 2022). Population controls are defined as any individuals without a history of COVID-19 (Chen X. et al., 2022). The GWAS dataset of COVID-19 hospitalization compares hospitalized COVID-19 cases (*n* = 9,986) with population controls (*n* = 1,877,672). In the dataset, hospitalized COVID-19 cases are defined as hospitalized patients with COVID-19 (Chen X. et al., 2022). Population controls are defined as any individuals without hospitalization experience for COVID-19 (including individuals without COVID-19) (Chen X. et al., 2022). The GWAS dataset of COVID-19 severity compares severe COVID-19 cases (*n* = 5,870) with population controls (*n* = 1,155,203). In the dataset, severe COVID-19 cases are defined as hospitalized individuals with COVID-19 who required respiratory support (such as intubation, continuous positive airway pressure, bilevel positive airway pressure, etc.,) (Cui and Tian, 2021). Population controls are defined as any individuals without several COVID-19 (including individuals without COVID-19) (Cui and Tian, 2021).

### 2.3 Statistical analysis

In the current MR study, a random-effect inverse variance weighted (IVW) model was carried out to verify the causal influence of PA intensity (light and moderate-to-vigorous intensity) on COVID-19 susceptibility, hospitalization, and severity (Burgess et al., 2017). The IVW model can offer a pooled causal estimate by combining the Wald ratio of each SNP on the outcome (Chen X. et al., 2022). The results of the IVW model were presented as odds ratios (OR) with corresponding 95% confidence intervals (CI). The statistical power of SNPs was calculated by an online tool available at http://cnsgenomics.com/shiny/mRnd/ (Brion et al., 2013). Considering multiple testing, Bonferroni-correction was used for setting significance level (Larsson et al., 2020). A *p*-value <0.008 (0.05/2 exposures/3 outcomes) was considered statistically significant, and a *p*-value between 0.008 and 0.05 was considered suggestive evidence for a causal association (Larsson et al., 2020). Finally, four sensitivity tests were performed as follows: 1) The MR-Egger intercept test was used to assess the directional horizontal pleiotropy, a major threat to the IVW estimator (Bowden et al., 2015). This intercept test can assess the average horizontal pleiotropy of all IVs under the “InSIDE” assumption. An intercept not significantly different from 0 is the evidence of no directional horizontal pleiotropy, otherwise, there can be a directional horizontal pleiotropy or the “InSIDE” assumption is violated (or both) (Burgess and Thompson, 2017); 2) Funnel plots were also used for assessing directional horizontal pleiotropy, and a symmetrical distribution is evidence of no directional pleiotropy; 3) The MR-PRESSO test was also used for assessing horizontal pleiotropy, which evaluates the overall horizontal pleiotropy amongst all IVs in a single MR test by comparing the observed distance of all the variants to the regression line (residual sum of squares) to the expected distance under the null hypothesis of no horizontal pleiotropy (Verbanck et al., 2018). The MR-PRESSO can also re-evaluate the association based on IVW after removing pleiotropic SNPs; 4) Cochran’s Q statistic was used to assess the degree of heterogeneity across the individual effect estimates derived from every genetic variant (Liu et al., 2021) and a non-significant Q value may also to a certain extent imply the absence of horizontal pleiotropy issue (Hemani et al., 2018); 5) The Leave-One-Out (LOO) was used to examine if the pooled estimate is disproportionately influenced by each genetic variant (Liu et al., 2021). In the current study, all analyses were conducted using the TwoSampleMR package (version 0.5.6) in R software (version 4.2.1).

## 3 Results

### 3.1 MR analysis

#### 3.1.1 Light physical activity

Seven SNPs (rs10166518, rs11179465, rs12021614, rs1268539, rs647347, rs74800845, and rs9878906) were used as instrumental variables for light PA, and no bias of weak instrument was observed in the statistic power test (F-statistic >10). Table 1 shows the causal influence of genetically proxied light PA on COVID-19 susceptibility, hospitalization, and severity. According to the result of the IVW model (Table 1), light PA significantly reduced the risk of COVID-19 infection (OR = 0.644, 95% CI: 0.480–0.864, *p* = 0.003). However, only suggestive evidence indicated that light PA significantly reduced the risk of COVID-19 hospitalization (OR = 0.446, 95% CI: 0.227–0.879, *p* = 0.020) and severe complications (OR = 0.406, 95% CI: 0.167–0.446, *p* = 0.046).

**TABLE 1:** Results of the IVW model and statistic power.

Outcome	Intensity	OR (95% CI)	*p*	F-statistical
Susceptibility	Light PA	0.644 (0.480–0.864)	*p* = 0.003**	3,840
	M-V PA	1.243 (0.530–2.916)	*p* = 0.617	2,533
Hospitalization	Light PA	0.446 (0.227–0.879)	*p* = 0.020*	4,305
	M-V PA	1.987 (0.320–12.332)	*p* = 0.461	2,840
Severity	Light PA	0.406 (0.167–0.446)	*p* = 0.046*	2,648
	M-V PA	1.966 (0.334–11.559)	*p* = 0.455	1767

Note: PA; physical activity, M-V; moderate to vigorous, *; suggestive evidence (0.008 < *p* < 0.05), **; statistically significant (*p* < 0.008).

#### 3.1.2 Moderate-to-vigorous physical activity

Five SNPs (rs10067451, rs10880697, rs12041071, rs55938136, and rs6002268) were used as instrumental variables for moderate-to-vigorous PA, and no bias of weak instrumental was observed in the statistic power test (F-statistical >10). Table 1 shows the causal influence of genetically proxied moderate-to-vigorous PA on COVID-19 susceptibility, hospitalization, and severity. According to the results of the IVW model (Table 1), no statistically significant association was observed regarding the three COVID-19 outcomes.

### 3.2 Sensitively analysis

In the current MR analysis, we used four sensitive analyses to estimate the robustness of results as following.

#### 3.2.1 Horizontal pleiotropy

**FIGURE 1:**
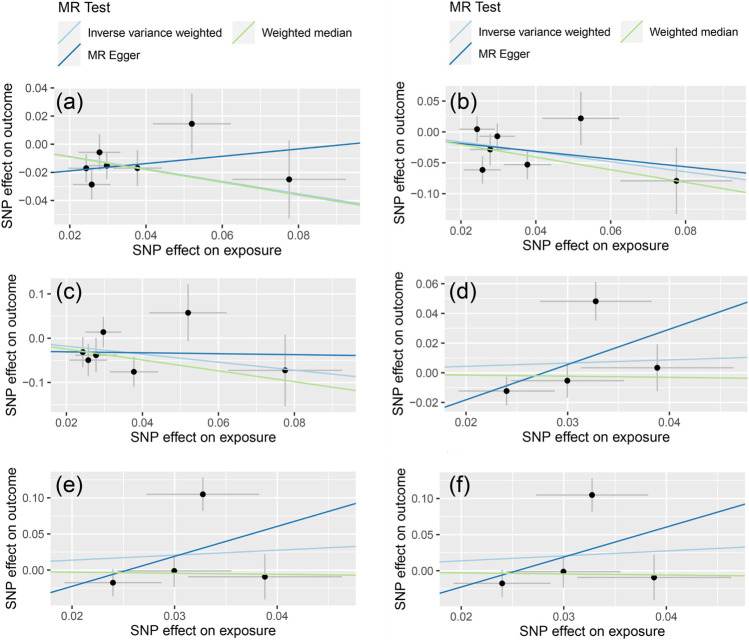
The results of MR-Egger regression [The **(A–C)** represent the causal influence of light PA on COVID-19 susceptibility, hospitalization, and severity respectively, the **(D–F)** represent the causal influence of moderate-to-vigorous PA on COVID-19 susceptibility, hospitalization, and severity respectively].

**FIGURE 2:**
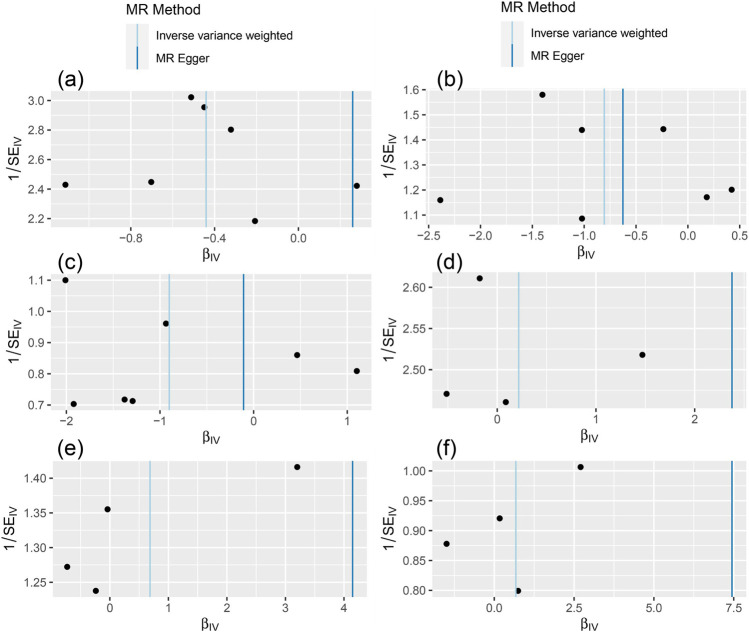
The results of funnel plot [The **(A–C)** represent the causal influence of light PA on COVID-19 susceptibility, hospitalization, and severity respectively, the **(D–F)** represent the causal influence of moderate-to-vigorous PA on COVID-19 susceptibility, hospitalization, and severity respectively].

The MR-Egger test was performed to assess the directional horizontal pleiotropy, and the results were demonstrated in Table 2; Figure 1. No significant directional horizontal pleiotropy was observed in all results (*p* > 0.05 and the intercept approximated zero). The funnel plots also showed low risks of directional horizontal pleiotropy regarding our IVW estimations (Figure 2). The MR-PRESSO detected no horizontal pleiotropy for the associations of light PA with COVID-19 infection, hospitalization, and severe complications. Some pleiotropic SNPs were found for other non-significant associations, but the re-analyses without those pleiotropic SNPs did not substantially change the results.

**TABLE 2:** Results of sensitively tests.

		Q statistic	MR-egger	Leave-one-out
Susceptibility	Light PA	*p* = 0.367	*p* = 0.165	No outliers
	M-V PA	*p* = 0.002*	*p* = 0.505	No outliers
Hospitalization	Light PA	*p* = 0.198	*p* = 0.878	Outliers
	M-V PA	*p* = 0.001*	*p* = 0.638	No outliers
Severity	Light PA	*p* = 0.401	*p* = 0.592	Outliers
	M-V PA	*p* = 0.046*	*p* = 0.283	No outliers

Note: PA; physical activity, M-V; moderate to vigorous, *; statistically significant (*p* < 0.05).

#### 3.2.2 Heterogeneity

The Cochran’s Q statistic was performed to assess the degree of heterogeneity, and the results were demonstrated in Table 2. No significant heterogeneity was observed regarding light PA results (*p* > 0.05). By contrast, significant heterogeneities were observed in the effects of moderate-to-vigorous PA on susceptibility (*p* = 0.002), hospitalization (*p* = 0.001), and severity (*p* = 0.046).

#### 3.2.3 Leave-one-out (LOO)

The LOO results were demonstrated in Table 2; Supplementary Figure S1. The causal influence of light PA on COVID-19 susceptibility was not substantially altered by any individual SNP. By comparison, the effects of light PA on the risk of COVID-19 hospitalization and severe complications were affected by a single SNP.

## 4 Discussion

As far as we know, this is the first study using large-sample GWAS data to investigate the causal influence of different PA intensities on COVID-19 susceptibility, hospitalization, and severity. Our finding indicates that genetically-proxied light PA may reduce COVID-19 susceptibility. Regarding COVID-19 hospitalization and severity, we found that genetically-predicted light PA, to a certain extent, reduced the risk of hospitalization and severity. These results, however, need to be interpreted with caution given their relaxed/uncorrected significance level. Furthermore, the sensitivity analysis (LOO) indicated that the effects of light PA on COVID-19 hospitalization and severity could be altered by a specific SNP. Considering these limitations, more studies are still necessary to clarify the role of light PA in affecting COVID-19 hospitalization and severity.

Regarding moderate-to-vigorous PA, our finding indicates that no causal influence of it on COVID-19 susceptibility, hospitalization, and severity. These findings were consistent with previous MR studies. Chen X. et al. (2022) performed an MR study to investigate the impact of self-reported moderate-to-vigorous PA on COVID-19 susceptibility, hospitalization, and severity, and observed similar results. Another MR study by Zhang et al. (2020) found a non-significant effect of moderate-to-vigorous PA on COVID-19 outpatient and deaths (Zhang et al., 2020). These findings collectively indicate that moderate-to-vigorous PA may not be a protective factor against COVID-19 events.

In general, our findings concerning the two PA intensities indicate that light PA may decrease the risk of COVID-19 infection, but the effect may disappear with increasing intensity. These findings partially support the J-theory that increasing PA intensity is associated with a higher risk of upper respiratory tract infections (Nieman, 1994; Matthews et al., 2002). However, it is notable that the J-theory highlights the effectiveness of moderate PA value, but not light PA (Matthews et al., 2002). The limited GWAS datasets we have may be a reason for this difference. Our dataset rudely divided PA intensity into two categories (light and moderate-to-vigorous intensity), which forbids us to specify the effect of moderate intensity. To offer more persuasive evidence, we recommend focusing on the dose-response relationship between the full range of PA intensity and COVID-19 outcomes.

From the epidemiological perspective, immune modulation is the fulcrum of most diseases, and COVID-19 is no exception (Cui and Tian, 2021). Currently, two major mechanisms have been proposed to explain the protective effect of PA against COVID-19 infection (Nigro et al., 2020), which may help explain our findings. First, PA can improve the function and action of tissue macrophages and promote the activation and recirculation of key immune system factors, and then strengthen the immune system (Nigro et al., 2020). These effects contribute to limiting viruses’ entry, translation, replication, and assembly (Jee, 2020; Diamond and Kanneganti, 2022). Second, PA can raise the level of salivary lactoferrin (one of the main antimicrobial proteins in saliva) (Woods et al., 2020). The lactoferrin secretion contributes to preventing DNA and RNA viruses from infecting cells, thereby reducing the risk of upper respiratory tract infections (Woods et al., 2020).

Although some distinctive causal associations were observed in this study, some limitations should be noted when interpreting our findings. First, our MR analysis only included European ancestry participants. Thus, our findings cannot be generalized to other populations. Second, only a few SNPs (*n* = 5 and 7) were used as instrumental variables, which might limit the power of the analyses. Furthermore, owing to the limited available GWAS data, the PA intensity was rudely divided into two categories (light and moderate to vigorous intensity), which may mask the true relationships between different PA intensities and COVID-19 outcomes. Thus, future study needs to consider extra or continuous intensity ranges when new GWAS data emerge.

## 5 Conclusion

The current MR study utilizes large sample GWAS datasets to examine the causal influence of light and moderate-to-vigorous PA on COVID-19 susceptibility, hospitalization, and severity. Despite some limitations, we provide genetic evidence that light PA may lower the risk of COVID-19 infection. To provide more scientific and quantitative evidence, we call for future research to focus on the full range of PA intensity when new GWAS datasets emerge.

## Data Availability

The raw data supporting the conclusion of this article will be made available by the authors, without undue reservation.
