# Supplementary material for: The causal effect of physical activity intensity on COVID-19 susceptibility, hospitalization, and severity: Evidence from a mendelian randomization study
Source: Front Physiol. 2023 Mar 8;14:1089637. doi: 10.3389/fphys.2023.1089637 (PMC10030504; doi:10.3389/fphys.2023.1089637)
Supplement: Supplementary file 1 [file DataSheet1.DOCX]

Supplementary Material

# Supplementary Figures and Tables

**Table S1.** Instrument variables of light PA

| SNP | Effect_allele | Other_allele | Beta | Se | P_value |
| --- | --- | --- | --- | --- | --- |
| rs10166518 | C | T | 0.025755 | 0.004965 | 2.14E-07^***^ |
| rs11179465 | G | T | -0.07755 | 0.014943 | 2.10E-07^***^ |
| rs12021614 | C | T | -0.0278 | 0.005459 | 3.54E-07^***^ |
| rs1268539 | C | A | -0.02972 | 0.004727 | 3.24E-10^***^ |
| rs647347 | A | G | 0.024308 | 0.004763 | 3.33E-07^***^ |
| rs74800845 | G | A | -0.05202 | 0.01022 | 3.57E-07^***^ |
| rs9878906 | A | C | 0.037749 | 0.006344 | 2.67E-09^***^ |

**Note:** SNP; Single nucleotide polymorphisms, ***; significance threshold *p* < 5×10^-7^.

**Table S2.** Instrument variables of moderate to vigorous PA

| SNP | Effect_allele | Other_allele | Beta | Se | P_value |
| --- | --- | --- | --- | --- | --- |
| rs10067451 | G | A | 0.038802 | 0.007511 | 2.39E-07^***^ |
| rs10880697 | G | C | -0.02464 | 0.004852 | 3.78E-07^***^ |
| rs12041071 | A | G | -0.02996 | 0.005567 | 7.34E-08^***^ |
| rs55938136 | A | G | -0.03276 | 0.005481 | 2.28E-09^***^ |
| rs6002268 | T | C | 0.023991 | 0.004739 | 4.13E-07^***^ |

**Note:** SNP; Single nucleotide polymorphisms, ***; significance threshold *p* < 5×10^-7^.

## Supplementary Figures


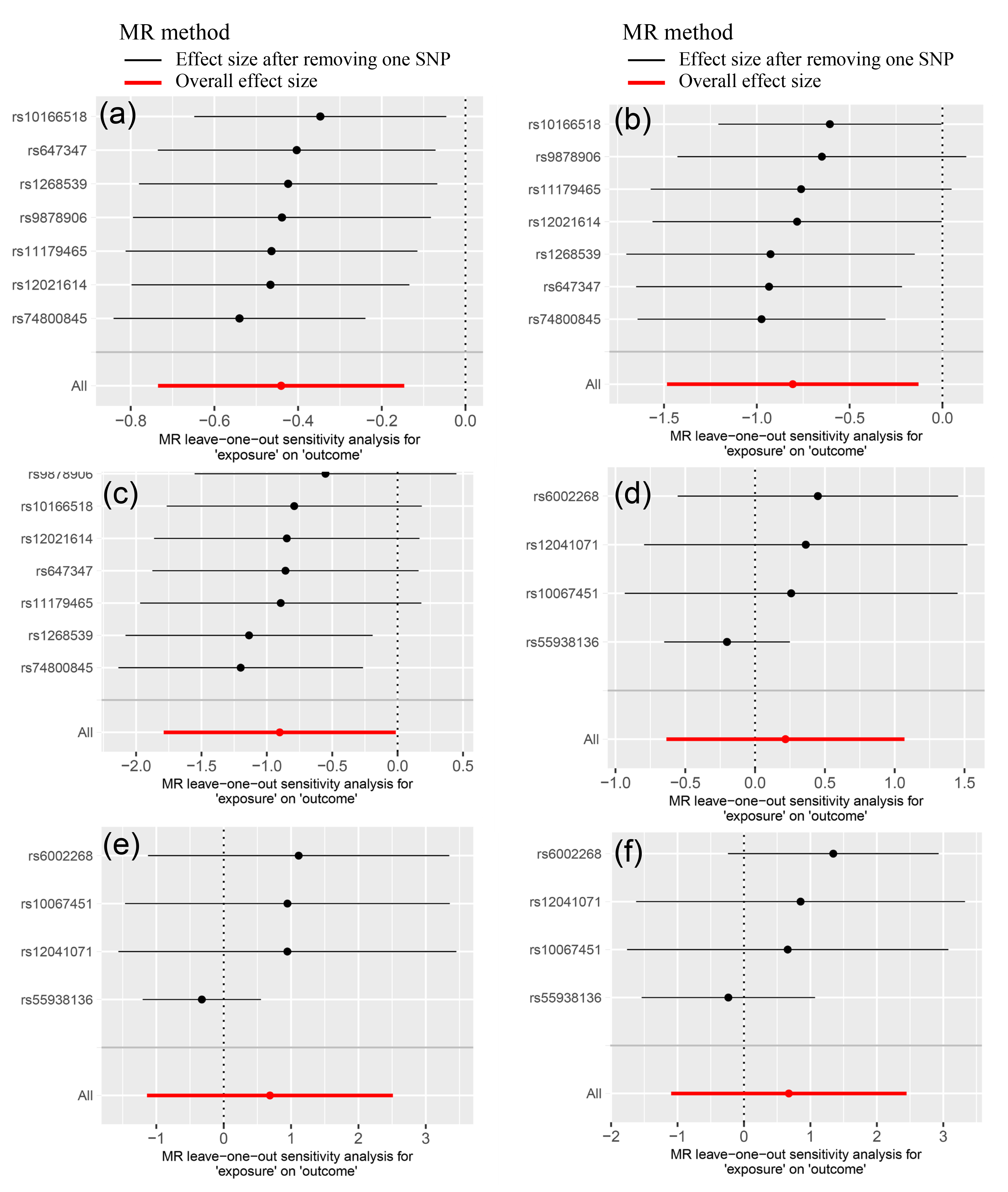


**Figure S1.** The results of Leave-One-Out(The a, b, and c represent the causal influence of light PA on COVID-19 susceptibility, hospitalization, and severity respectively, the d, e, and f represent the causal influence of moderate to vigorous PA on COVID-19 susceptibility, hospitalization, and severity respectively).
